# Is an insecure job better for health than having no job at all? A systematic review of studies investigating the health-related risks of both job insecurity and unemployment

**DOI:** 10.1186/s12889-015-2313-1

**Published:** 2015-09-29

**Authors:** Tae Jun Kim, Olaf von dem Knesebeck

**Affiliations:** Department of Medical Sociology, University Medical Centre Hamburg-Eppendorf, Martinistrasse 52, 20246 Hamburg, Germany

## Abstract

**Background:**

Though previous research repeatedly found that being employed is better for health than having no job at all, evidence suggests that employment is not always beneficial for health. With especially job insecurity reflecting a contemporary health risk for the employed, a systematic review was performed to assess if insecure employment can be as detrimental for health as unemployment, and to determine whether these associations vary according to different health measures and among men and women.

**Methods:**

The literature search was conducted in the databases Medline, Embase and PsychInfo. In order to allow a more accurate comparison between the two risk factors, studies were included if the data for job insecurity and unemployment was ascertained from the same sample, and contained a quantitative analysis for both exposures towards one (or more) health outcome(s).

**Results:**

Out of 375 articles, in total, 13 studies were included in the systematic review. In 24 analyses contrasting the health-related associations between job insecurity and unemployment, 16 statistically significant associations were found for each exposure. According to the different health outcomes used, job insecurity and unemployment were strongly related to mental health, whereas job insecurity was more strongly associated with somatic symptoms. Unemployment showed stronger relations with worse general health and mortality. In 4 out of 16 gender-stratified analyses, significant associations between job insecurity/unemployment and health were found for men but not for women. Beyond that, associations were significant or insignificant in both gender groups.

**Conclusions:**

Though there were moderate differences across the health outcomes, overall, it was found that job insecurity can pose a comparable threat to health than unemployment. Policy interventions should therefore not only consider health risks posed by unemployment, but should also aim at the reduction of insecure employment.

**Electronic supplementary material:**

The online version of this article (doi:10.1186/s12889-015-2313-1) contains supplementary material, which is available to authorized users.

## Background

The influence of unemployment on health was investigated in numerous studies. It was consistently found that joblessness is linked to poorer self-rated health, mental illness, more physical complaints, an increased risk for coronary heart diseases and higher all-cause mortality [1–5]. Hereafter, unemployment is recognized as a crucial hazard, whereas paid work is generally known to be potentially health promoting, since it offers financial security, daily time structures and social inclusion as well as the development of personal identities [6].

While the introduction of a flexible labor-market was initially regarded as an answer to joblessness, studies, however, showed that having any job is not always better than having no job [7–9]. Even though flexible employment relations were accompanied by a major decline of physically dangerous occupations, the simultaneous growth of service-based industries was associated with new psychosocial risks [10], indicating that poor psychosocial working conditions might detriment health to a similar degree when compared to unemployment [11, 12]. Of these, particularly insecure employment constitutes a major work-related stressor that is related to poor self-rated health, increased psychiatric morbidity, high cholesterol, hypertension, and increased incident coronary heart disease [13–16]. Therefore, job insecurity does not only yield a risk for public health, but also concerns a growing number of employees, affecting even the insecure employed that do not necessarily experience future job loss. And with the labor markets becoming increasingly insecure, new challenges to the broader population are posed. In contrast to the actual experience of job loss, job insecurity defines a perceptual phenomenon [17], including the everyday experience of a prolonging uncertainty of the future [18]. This definition of job insecurity should be distinguished from other objective ascertainments of insecure employment, for example temporal employment (fixed-term, part-time) or former fragmented working careers. The current study aims to explore if the subjective threat of losing one’s job can affect health similarly to unemployment. We systematically reviewed the literature by focusing on studies that simultaneously investigated job insecurity and unemployment for a broader range of health outcomes. In contrasting the subsamples of the insecure employed and unemployed within a respective study we addressed the following questions: How strong and consistent are the associations between perceived job insecurity and unemployment with health? Do the associations vary depending on the diverse health measures that were used in studies? And since recent studies found that the associations between psychosocial work characteristics, unemployment and health are also influenced by gender [19–26], we additionally examined if the relationship between job insecurity, unemployment and health varies among men and women.

## Methods

### Search strategy and inclusion criteria

The literature search was conducted on 2^nd^ of March 2015. In order to provide insights to the traceability and reproducibility of our findings, this review was performed on the basis of the PRISMA guidelines [27]. A checklist containing the PRISMA guidelines for reporting systematic reviews is available as an appendix (see Additional file 1). Since no other review focused on a simultaneous analysis of job insecurity and unemployment, no restrictions on the publication date were considered. For the screening of potentially eligible studies, the computerized databases MEDLINE, EMBASE and PSYCHINFO were explored for peer-reviewed articles that enclosed the following keywords for job insecurity, unemployment and health: (“job insecurity” OR “job instability” OR “insecure employment” OR “insecure job*” OR “insecure work” OR “job security” OR “job stability” OR “secure employment” OR “secure job*” OR “job uncertainty”) **AND** (unemployment OR “job loss” OR joblessness) **AND** (health OR “quality of life” OR well-being OR wellbeing OR mortality OR morbidity OR disease* OR illness* OR sickness).

To respond to design-related heterogeneity that is usually found in the comparison of observational studies [28], data on job insecurity and unemployment had to be derived from the same study to minimize bias and control for sample specific characteristics. Additionally, identified records had to entail a quantitative analysis of job insecurity and unemployment with one or more health outcomes. Furthermore, the measurement of job insecurity had to be based on self-reports, since an objective measure (e.g. contractual insecurity or fragmented working careers), does not adequately reflect job insecurity as an involuntarily and subjective stressor [29]. Studies were also excluded if they measured non-employment instead of unemployment, to eliminate those who have not yet entered the working force (students, trainees) or who have left due to retirement.

### Data extraction

Descriptive characteristics of studies entailed information on author, country (region), research design, study year, follow-up duration (if appropriate), sample size, response rate, mean age, gender distribution and considered confounders. Additionally, the measurement of health outcomes, job insecurity and unemployment were assessed. For the comparison, results for both risk factors were contrasted for each study by focusing on the most informative statistical measurement. In order to gain additional insights on differences between men and women, gender stratified results were extracted from the articles, if available. Though the reference to scientific data analyses may have led to a better estimation of the risk of both perceived job insecurity and unemployment, we decided not to perform a meta-analysis for a couple of reasons: First, number of included studies was rather small. Second, studies substantially varied in their study designs (cross-sectional vs. longitudinal studies), their sampling procedures and data collection methods. Third, longitudinal studies were also characterized by large differences in their observation period and the measurement of predictors and outcomes varied across all included studies. Finally, substantial variations in the consideration of confounders were evident, and statistical analyses and the reporting of effect sizes differed from study to study.

## Results

### Literature search

**Fig. 1:**
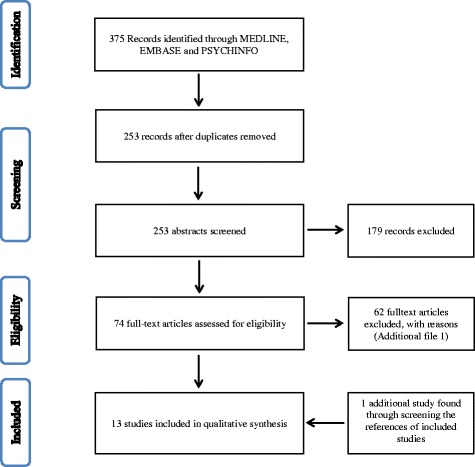
Selection and inclusion of studies (PRISMA Flow Diagram)

The screening, exclusion and inclusion process was performed on basis of the PRISMA Flowchart (Fig. 1). A total of 375 publications were identified through the databases Medline, Embase and PsychInfo. After removing duplications, 253 records remained. After abstract screening, another 179 studies were deselected since they did not address the subject of job insecurity and unemployment in its relation to health. Out of the remaining 74 records that were assessed in full-text and tested for eligibility, 62 articles were excluded for several reasons (see Additional file 2). By screening the grey literature and references of included studies, one additional record was found [30]. Finally, 13 articles were included in the systematic review.

### Overview of included studies

Table 1 summarizes the methodological characteristics of the 13 included studies published between 1997 and 2013. Most of the studies were conducted in Germany and USA. The study samples (total *N* = 91,085) varied greatly in size. 46 % of the overall data was derived from the USA, 19.8 % from North- and West-Europe, 18.8 % from Russia and 15.3 % from Australia. The mean age of participants included in this review ranged from 29 to 54.8 (excluding four studies in which respective information was not available), whereas only three studies had a mean age lower than 36. In three studies, men were overrepresented. The remaining 9 studies did not reveal major differences towards the distribution of gender, and information was not available for one study.

**Table 1:** Methodological characteristics of included studies

Author (year) Country	Research design (specific population)	Study year (follow-up)	Sample size	Baseline resp. (follow-up rate)	Age mean	Male in %	Covariates in adjusted multivariable model
Amick, 2002 USA [40]	Cohort study (working cohort)	1968 (24 years)	25,413	n.a. (30.5 %)	45.0	44.9	Age, race, gender, year, family income, family size, retirement, unemployment, retirement by age interaction, race by age interaction, baseline disability, job strain
Berth, 2003 GER [35]	Cross-sectional study	2002 (−−)	420	72.0 % (−−)	29.0	46.8	--
Berth, 2005 GER [39]	Cross-sectional study	2003 (−−)	419	71.0 % (−−)	30.1	46.1	--
Burgard, 2009 USA [36]	Two complementary cohort studies	1986 (3 years) 1995 (10 years)	1,867	70.0 % (87.0 %)	41.2	53.6	Age, gender, race, marital status, household income, education, job insecurity, involuntarily job loss, employed at follow-up, self-employed, part-time, health shock, high blood pressure, neuroticism, smoking status, self-rated health and depressive symptoms at baseline
			1,712	61.0 % (80.0 %)	43.4	43.7	
Ferrie, 1997 GB [31]	Cohort study (subsample Whitehall-II)	1985 (9 years)	666	73.0 %^a^ (81.2 %)	n.a.	76.7	Age, grade and baseline value of the variable
Flint, 2013 GB [32]	Cohort study	1991 (16 years)	10,494	92.0 %^b^ (66.2 %)	n.a.	48.4	Age, age^2^, education, physical health problems, spousal joblessness, spousal GHQ-12, marital status, unemployed spells in past 12 months, residence in social housing, substance abuse, equivalised household income, permanent sickness
Green, 2011 AUS [33]	Cohort study	2001 (7 years)	13,969	93.5 % (93.3 %)	36.1	n.a.	Age, marital status, number of children, education, income, Employability if unemployed, re-employment difficulty, personal characteristics (extroversion, conscientiousness, emotional stability, openness to experience), long term health condition, others present in interview, regional Australia, remote Australia
Levenstein, 2001 USA [15]	Cohort study	1965 (29 years)	6,928	86.2 % (39.4 %)	n.a.	43.7	Age, gender, ethnicity, educational status, occupational status, not in labor force, depression and anomy score, BMI, smoking and alcohol consumption, leisure time physical activity, having had a medical checkup within 2 years before the follow-up study.
Mandal 2011 USA [30]^c^	Cohort Study	1992 (14 years)	5994	81.6 (88.6 %)	54.8	48.8	Age, gender, ethnicity, educational level, suffered business closure, displaced x expectation, got married/partnered, got separated/divorced/widowed, change in housing assets, job tenure years, type of occupation, S&P 500 returns
Makikangas, 2011 FIN [34]	Cohort study (Finnish managers)	1996 (10 years)	1,035	64.0 % (38.8 %)	41.9	95.0	--
Mewes, 2013 GER [37]	Cross-sectional study	2007 (−−)	2,510	61.9 % (−−)	42.0	45.5	--
Perlman, 2009 RUS [41]	Cohort study	1994 (9 years)	17,154	88.8 % (59.6 %)	n.a.	52.6	Age, education, occupation, alcohol, smoking, material goods, age at entry, district in Russia, and cluster by household.
Zenger, 2013 GER [38]	Cross-sectional study	2010 (−−)	2,504	56.2 % (−−)	51.8	46.7	--

^a^Since the response rate for the subsample of PSA-respondents was not available, the overall response-rate for the Whitehall-II sample was used; ^b^Data on the follow-up rate were looked up in the manual of the British Household Panel Survey (Taylor et al. 2010 [42]); ^c^Pooled results for age-groups 45–54 and 55–65

Abbreviations: *BMI* Body-Mass-Index; *GHQ* General health Questionnaire; *S&P 500* Standard and poor’s 500 stock market index

According to the health outcomes, measures of mental health were most frequently used. Four studies assessed mental health by either recurring to the General Health Questionnaire (GHQ) [31, 32], the Short Form Health Survey (SF-36) [33] or job-related affective well-being, the ladder including dimensions of anxiety, depression, comfort and enthusiasm [34]. Depression was either measured with the subscale of the Hospital Anxiety and Depression scale (HADS) [35], the Center for Epidemiological Studies Depression Scale (CES-D) [30, 36] or with the Patient Health Questionnaire (PHQ) [37, 38]. Anxiety was also ascertained with the subscale of HADS [35] and PHQ [37, 38]. Other mental health related indicators were fatigue, measured with the Gießener Beschwerdebogen (GBB) [35], and somatoform disorders, measured with the PHQ [37] as well as psychological distress, ascertained with the Symptom Checklist-9 (SCL-9) [35]. Moreover, five studies examined associations between perceived job insecurity, unemployment and general health. Three studies ascertained self-rated health on a 5-point scale [31, 35, 36, 39], while a single study assessed the individual’s satisfaction with one’s own health [39]. Other outcomes of general health were long-standing illness and number of health problems [31]. Furthermore, a total of four studies assessed somatic symptoms as a health outcome. One study focused on stomach trouble, joint pain, heartache and an index of complaints measured by the GBB [35]. Another study examined the reported frequency of somatic complaints through the PHQ [38], while two studies either ascertained the number of symptoms [31] or hypertension [15]. Mortality was used as a health outcome in two studies [40, 41]. It has to be mentioned that of the included 13 studies, 5 studies investigated two or more health indicators. In terms of unemployment, three measures were differentiated: (1) former job loss experience, (2) duration of unemployment and (3) actual unemployment.

### Major findings for different health outcomes

In 24 analyses that compared the health-related risks between the subsamples of the insecure employed and unemployed within each study, 16 statistically significant associations were found for job insecurity and unemployment, respectively (Table 2).

**Table 2:** Associations between job insecurity, unemployment and health

No.	Health dimension	Health indicator	Publication	Unemployment measurement	Statistics	Job insecurity	Unemployment
1.	General health	Self-rated health (5-point scale)	Berth 2003 [35]	Job loss experience	*F*-value (p)	1.40 (n.s.)	6.92 (<.05)
2.		Satisfaction with one’s own health (5-point scale)	Berth 2005 [39]	Job loss experience	Mean (p)	6.24 (<.01)	6.69 (<.05)
3.		Self-rated health (5-point scale)	Burgard 2009 (Cohort 1) [36]	Job loss experience	Unstandardized OLS coefficient (p)	−0.032 (n.s.)	0.032 (n.s.)
4.		Self-rated health (5-point scale)	Burgard 2009 (Cohort 2) [36]	Job loss experience	Unstandardized OLS coefficient (p)	−0.039 (n.s.)	−0.005 (n.s.)
5.	Mental health	Mental Health (GHQ)	Flint 2013 [32]	Current employment status	Unstandardized OLS coefficient (p)	1.11 (<.05)	2.21 (<.05)
6.		Job-related affective well-being	Makikangas 2011 [34]	Job loss experience	Odds Ratio (p)	6.32 (<.05)	4.86 (<.05)
7.		Anxiety (HADS)	Berth 2003 [35]	Job loss experience	*F*-value (p)	10.21 (<.001)	6.74 (<.01)
8.		Anxiety (PHQ-7)	Mewes 2013 [37]	Current employment status	Mean (p)	2.6 (<.001)	2.6 (<.001)
9.		Anxiety (PHQ-4)	Zenger 2013 [38]	Current employment status, job loss experience, unemployment duration	*F*-value (p)	15.45 (<.001)	Frequency: 24.29 (<.001)
							Duration: 23.90 (<.001)
							Status: 4.90 (<.001)
10.		Depression (HADS)	Berth 2003 [35]	Job loss experience	*F*-value (p)	17.91 (<.001)	10.68 (<.001)
11.		Depressive symptoms (CES-D)	Burgard 2009 (Cohort 1) [36]	Job loss experience	Unstandardized OLS coefficient (p)	−0-094 (n.s.)	0.042 (n.s.)
12.		Depressive symptoms (CES-D)	Burgard 2009 (Cohort 2) [36]	Job loss experience	Unstandardized OLS coefficient (p)	0.053 (n.s.)	0.020 (n.s.)
13.		Depressive symptoms (CES-D)	Mandal 2011 [30]	Job loss experience	Unstandardized OLS coefficient (p)	−0-036 (n.s.)	0.329 (<.05)
14.		Depressive disorder (PHQ-9)	Mewes 2013 [37]	Current employment status	Mean (p)	2.9 (<.001)	3.6 (<.001)
15.		Depression (PHQ-4)	Zenger 2013 [38]	Current employment status	*F*-value (p)	14.24 (<.001)	Frequency: 35.11 (<.001)
							Duration: 41.74 (<.001)
				Job loss experience			Status: 7.04 (<.001)
				Unemployment duration			
16.		Somatoform disorder (PHQ-15)	Mewes 2013 [37]	Current employment status	Mean (p)	3.7 (<.001)	3.9 (<.001)
17.		Psychological distress (SCL-9)	Berth 2003 [35]	Job loss experience	*F*-value (p)	3.49 (<.05)	3.29 (<.01)
18.		Fatigue (GBB)	Berth 2003 [35]	Job loss experience	*F*-value (p)	5.70 (<.01)	2.64 (n.s.)
19.	Mortality	Mortality	Amick 2002 [40]	Current employment status	Odds Ratio (p)	0.95 (n.s.)	2.26 (<.05)
20.	Somatic symptoms	Stomach trouble (GBB)	Berth 2003 [35]	Job loss experience	*F*-value (p)	2.98 (<.05)	2.03 (n.s.)
21.		Joint pain (GBB)	Berth 2003 [35]	Job loss experience	*F*-value (p)	2.81 (<.05)	2.13 (n.s.)
22.		Heartache (GBB)	Berth 2003 [35]	Job loss experience	*F*-value (p)	2.21 (n.s.)	1.84 (n.s.)
23.		Index of complaints (GBB)	Berth 2003 [35]	Job loss experience	*F*-value (p)	5.14 (<.01)	3.46 (<.05)
24.		Complaints (PHQ-4)	Zenger 2013 [38]	Current employment status, job loss experience, unemployment duration	*F*-value (p)	5.93 (<.01)	Frequency: 6.02 (<.01)
							Duration: 10.99 (<.001)
							Status: 4.18 (<.001)

Abbreviations: *CES-D* Center for Epidemiological Studies Depression Scale; *GBB* Gießener Beschwerdebogen; *GHQ* General health Questionnaire; *HADS* Hospital Anxiety and Depression Scale; *n.s.* not significant; *OLS* Ordinary least squares; *p* p-value, *PHQ* Patient Health Questionnaire; *SCL-9* Symptom Checklist-9

In four studies that examined general health, stronger, but inconsistent relations were found for unemployment. In the 14 analyses that investigated the outcome mental health, overall, strong associations were evident for both risk factors. For mortality, a single study found strong and statistically significant associations with unemployment, but not with job insecurity. Concerning the analysis of somatic symptoms, three out of 5 studies found statistically significant relations with job insecurity, whereas only one study found associations with unemployment. The additional differentiation of the measurement of job loss revealed that the unemployment frequency and duration showed stronger associations than the actual unemployment status.

The health-related risks of job insecurity and unemployment for men and women are summarized in Table 3. With regards to the 6 analyses on general health and long-standing illness no statistically significant associations were found for either job insecurity or unemployment among men and women. In the four analyses that focused on the relationship with mental health, no to small differences between men and women emerged. In one study on mortality, statistically significant associations were only found for unemployment, but not for job insecurity. However, this relationship could only be found for unemployed men. In terms of somatic symptoms, statistically significant relations of hypertension with job insecurity and unemployment were only found for men.

**Table 3:** Gender stratified associations between job insecurity, unemployment and health

No.	Health dimension	Health outcome	Publication	Statistics	Job insecurity		Unemployment	
					Men	Women	Men	Women
1.	General health/illness	Long-standing illness	Ferrie 1997 [31]	Odds Ratio (p)	1.06 (n.s.)	3.39 (n.s.)	1.62 (n.s.)	3.76 (n.s.)
2.		Number of health problems (over the last year)	Ferrie 1997 [31]	Mean (p)	1.34 (n.s.)	2.39 (n.s.)	1.57 (n.s.)	2.03 (n.s.)
3.		Self-rated health (5-point scale)	Ferrie 1997 [31]	Odds Ratio (p)	1.52 (n.s.)	1.40 (n.s.)	1.54 (n.s.)	2.08 (n.s.)
4.	Mental health	Mental Health (GHQ)	Ferrie 1997 [31]	Mean (p)	2.63 (<.001)	2.82 (<.05)	2.29 (<.01)	2.57 (n.s.)
5.		Mental Health (SF-36)	Green 2011 [33]	Unstandardized OLS coefficient (p)	−5.113 (<.001)	−3.137 (<.001)	−8.037 (<.001)	−8.422 (<.001)
6.	Mortality	Mortality	Perlman 2009 [41]	Hazards ratio (p)	0.99 (n.s.)	1.15 (n.s.)	1.39 (<.05)	0.67 (n.s.)
7.	Somatic symptoms	Number of symptoms (in the past fortnight)	Ferrie 1997 [31]	Mean (p)	3.63 (n.s.)	4.47 (n.s.)	3.94 (n.s.)	3.60 (n.s.)
8.		Hypertension	Levenstein 2001 [15]	Odds Ratio (p)	1.6 (<.05)	1.0 (n.s.)	2.7 (<.05)	0.8 (n.s.)

Abbreviations: *GHQ* General health Questionnaire; *n.s.* not significant; *OLS* Ordinary least squares; *p* p-value; *SF-36* Short Form (36) health survey

## Discussion

### Summary of findings

The aim of this study was to systematically analyze associations of job insecurity and unemployment with health. This is the first systematic review of studies that simultaneously analyze the health risks of job insecurity and unemployment. Although the results for job insecurity and unemployment showed some inconsistencies, it was revealed that both exposures represent significant (work-related) stressors that are associated with impaired health. In summary, the comparison of the two subsamples of the insecure employed and unemployed within studies suggested that job insecurity might be as harmful to health as unemployment, though differences were found for different health outcomes. In this regard, perceived job insecurity was slightly stronger associated with somatic symptoms, whereas stronger relations with unemployment were found for worse general health and an increased mortality risk. For mental health, strong but partly inconsistent associations were found for both risk factors. It has to be considered that the associations were generally stronger for respondents who were currently unemployed or were exposed to longer durations of joblessness. Likewise, former studies have also found that the impact of job insecurity increases over time. Longitudinal studies concentrating on chronic job insecurity and health [13, 43] indicate that job insecurity constitutes a chronic stressor which does not immediately affect health, but its impact intensifies on temporal expansion. Especially with respect to the framework of Lazarus and Folkman [44], persistent job insecurity presents a continuous source of uncertainty, in which the ongoing exposition to stress can ultimately impair health [45].

According to gender-specific health risks through job insecurity and unemployment, 9 of 16 analyses revealed statistically insignificant associations between job insecurity, unemployment and health among both genders. In three further cases, statistically significant associations were found that were similar among men and women. In the remaining analyses, associations were significant among men but not among women. This was especially true for mortality and a somatic symptom (hypertension). Generally speaking, the differences between men and women seem to be inconsistent, with insecurely employed or unemployed men having slightly higher risks for impaired health.

### Limitations

Though this was the first review to systematically examine relations between job insecurity, unemployment and ill health, several limitations on the interpretation of our study results should be considered. First, and even though we systematically searched databases for eligible studies, a risk of potentially missing out relevant articles still remains. We attempted to partly reduce this bias by screening the grey literature and references of included studies for additional (possibly) relevant articles. However, only one additional record was found as only a few studies included both job insecurity and unemployment in their analyses. Second, an extensive summary of a variety of studies certainly yields a risk of bias, since observational studies tend to substantially differ along their study-designs and methodological characteristics. Rather than assessing the unique quality of studies, we decided to focus on the comparison between job insecurity and unemployment within each study to minimize bias and control for these sample-specific characteristics. And although the calculation of an overall effect size may have led to a more precise illustration, a meta-analysis was not considered since the rather small number of included studies varied substantially in their study designs, statistical procedures and use of outcomes. Third, the generalizability of results on the relationship between job insecurity, unemployment and health is limited by the fact that only studies were included that considered both job-related indicators within their analyses. Fourth, health selection effects in the analysis of unemployment remain a major source of bias, since it includes the possibility that persons became unemployed due to their experience of poor health, rather than becoming ill through joblessness. Particularly in cross-sectional studies this scenario is likely to occur. As a consequence, especially the comparison of the health outcome somatic symptoms must be interpreted with caution, since all studies were cross-sectional in design. It has to be mentioned, though, that these risks do not only concern cross-sectional studies as the ascertainments of perceived job insecurity and unemployment in longitudinal studies do not fully cover potential changes during the observation period. As such, no distinction can be made if persons who were unemployed at the baseline survey have found a new job in the meantime or transitioned between unemployment and re-employment over the timeframe. This potential bias is also applicable to the measurement of perceived job insecurity. Fifth, publication bias figures to remain a major issue in systematic reviews that may lead to an overestimation of the associations between job insecurity, unemployment and health; thus further limiting our results. Finally, the fairly small number of studies for some health outcomes threatens the robustness of our findings.

## Conclusions

Despite these limitations, we found that perceived job insecurity and unemployment reflect independent stressors that may constitute major threats for public health. These associations varied along the use of different health outcomes. Both job insecurity and unemployment were strongly related to mental health. And while job insecurity was stronger associated with somatic symptoms, unemployment showed increased risks for worse general health and mortality. Our results imply that the anticipation of a (potential) job loss is similarly associated with worse health than the actual experience of unemployment. Given that an increasing number of employees are likely to experience their jobs as insecure in the future [46], different public health interventions are necessary to encounter the cumulative health consequences of job loss and insecure employment appropriately. For this, initial insecure employment or unemployment do not only affect subsequent health, as less healthy workers are also likely to end up in less secure jobs and thus promoting the risk for subsequent unemployment. Though labor market policies focusing on increasing flexible employment relations may result in short-term economic benefits, results suggest that insecure employment and job loss may entail increasing long-term consequences for individual and public health, economic productivity and the costs of the health care system. Thus, policies should not only focus on the health risks posed by unemployment, but should also aim at the reduction of job insecurity of the employed.

## Additional files

Additional file 1:
**PRISMA 2009 Checklist.** (DOC 63 kb) Additional file 2:
**Reasons for exclusion after fulltext screening.** (DOCX 29 kb)
