# Supplementary material for: Is an insecure job better for health than having no job at all? A systematic review of studies investigating the health-related risks of both job insecurity and unemployment
Source: BMC Public Health. 2015 Sep 29;15:985. doi: 10.1186/s12889-015-2313-1 (PMC4589035; doi:10.1186/s12889-015-2313-1)
Supplement: Additional file 2: — Reasons for exclusion after fulltext screening. (DOCX 29 kb) [file 12889_2015_2313_MOESM2_ESM.docx]

**Additional File 2 – Reasons for exclusion after fulltext screening**

|  | **Article** | **Reasons for Exclusion** |
| --- | --- | --- |
|  | Arango-Lasprilla JC, Ketchum JM, Gary KW et al. (2009) The Influence of Minority Status on Job Stability After Traumatic Brain Injury. PM and R, 41–49. | No health outcome |
|  | Asvall JE and Taipale V (1998) Symposium on Labour market changes and job insecurity: A challenge for social welfare and health promotion, Kellokoski, Finland, June 1996: Foreword. World Health Organization Regional Publications - European Series. | No quantitative analysis of both job insecurity AND unemployment towards the same health outcome |
|  | Arnetz BB, Brenner SO, Levi L, et al. (1991) Neuroendocrine and Immunologic Effects of Unemployment and Job Insecurity. Psychotherapy and Psychosomatics, Vol. 55(2-4). | Quantitative results were not tested for statistical significance |
|  | Bartley M (2012) Explaining health inequality: Evidence from the UK. Social Science and Medicine, 658–660. | No quantitative analysis of both job insecurity AND unemployment towards the same health outcome |
|  | Bartley M and Ferrie J (2001) Glossary: unemployment, job insecurity, and health. Journal of epidemiology and community health, 776–781. | No quantitative analysis of both job insecurity AND unemployment towards the same health outcome |
|  | Bartley M, Sacker A, Clarke P (2004) Employment status, employment conditions, and limiting illness: Prospective evidence from the British household panel survey 1991-2001. Journal of Epidemiology and Community Health, 501–506. | No quantitative analysis of both job insecurity AND unemployment towards the same health outcome |
|  | Beale N and Nethercott S (1987) [The health of industrial employees four years after compulsory redundancy]. The Journal of the Royal College of General Practitioners, 37(302), 390–394. | No health outcome |
|  | Beaune L, Morinis J, Rapoport A et al. (2013) Paediatric palliative care and the social determinants of health: Mitigating the impact of urban poverty on children with life-limiting illnesses. Paediatrics and Child Health (Canada), 181–183. | No quantitative analysis of both job insecurity AND unemployment towards the same health outcome |
|  | Berth H, Forster P, Balck F et al. (2008) Unemployment, job insecurity and the need for psychosocial support. Gesundheitswesen, 289–294. | No health outcome |
|  | Bhugra D and Minas IH (2007) Mental health and global movement of people. Lancet, 1109–1111. | No quantitative analysis of both job insecurity AND unemployment towards the same health outcome |
|  | Borg V, Kristensen TS, Burr H (2000) Work environment and changes in self-rated health: A five year follow-up study. Stress Medicine, 37–47. | No quantitative analysis of both job insecurity AND unemployment towards the same health outcome |
|  | Boscolo P, Di Gioacchino M, Reale M et al. (2011) Work stress and innate immune response. journal of immunopathology and pharmacology. Retrieved from http://ovidsp.ovid.com/ovidweb.cgi?T=JS&CSC=Y&NEWS=N&PAGE=fulltext&D=emed10&AN=21329566 | No quantitative analysis of both job insecurity AND unemployment towards the same health outcome |
|  | Boscolo P, Forcella L, Cortini M et al. (2012) Well-being at work and immune response. Advances in Neuroimmune Biology, 3–4. | No quantitative analysis of both job insecurity AND unemployment towards the same health outcome |
|  | Brenner SO and Levi L (1987) Long-term unemployment among women in Sweden. Social Science & Medicine, 25(2), 153–161. | No quantitative analysis of both job insecurity AND unemployment towards the same health outcome |
|  | Brenner SO and Starrin B (1988) Unemployment and health in Sweden: Public issues and private troubles. Journal of Social Issues, 44(4), 125–140. | No quantitative analysis of both job insecurity AND unemployment towards the same health outcome |
|  | Broom DH, D’Souza RM, Strazdins L et al. (2006) The lesser evil: Bad jobs or unemployment? A survey of mid-aged Australians. Social Science and Medicine, 575–586. | No explicit measurement of job insecurity (job insecurity summarized within multiple adverse working conditions) |
|  | Burchell B (1992) Towards a social psychology of the labour market: or Why we need to understand the labour market before we can understand unemployment. Journal of Occupational and Organizational Psychology, 65(4), 345–354. | No quantitative analysis of both job insecurity AND unemployment towards the same health outcome |
|  | Butterworth P, Leach LS, Strazdins L et al. (2011) The psychosocial quality of work determines whether employment has benefits for mental health: Results from a longitudinal national household panel survey. Occupational and Environmental Medicine, 806–812. | No quantitative analysis of both job insecurity AND unemployment towards the same health outcome |
|  | Cazals MP and Baubion-Broye A (1997) Social comparison and psychological well-being among young people in professionally precarious situations. [French]. Cahiers Internationaux de Psychologie Sociale, 52–60. | No quantitative analysis of both job insecurity AND unemployment towards the same health outcome |
|  | Cazals-Ferre MP and Llorca MC (2002) The impact of insecurity on individual vulnerability and health. [French]. Pratiques Psychologiques, 51–63. | No quantitative analysis of both job insecurity AND unemployment towards the same health outcome |
|  | Chastang F, Dupont L, Rioux P et al. (1998) The role of professional integration in suicidal recurrence. Annales de Psychiatrie, 241–247. | No health outcome |
|  | Chastang F, Rioux P, Dupont I et al. (1998) Suicide attempts and job insecurity: A complex association. European Psychiatry, 359–364. | No health outcome |
|  | Cooper CL (2007) Mental well being at work. Journal of Public Health, 131–132. | No quantitative analysis of both job insecurity AND unemployment towards the same health outcome |
|  | Curtin LL (1995) Job security: is nothing sacred anymore? Nursing management, 7–9. | No quantitative analysis of both job insecurity AND unemployment towards the same health outcome |
|  | De Witte (1999) Job insecurity and Psychological Well-being: Review of the literature and some unresolved issues. European Journal of Work and Organizational Psychology 8(2):155–177. | Comparison of two independent samples |
|  | Ferrie JE (1999) Health consequences of job insecurity. WHO regional publications, 59–99. | No quantitative analysis of both job insecurity AND unemployment towards the same health outcome |
|  | Ferrie JE, Shipley MJ, Marmot MG et al. (1995) Health effects of anticipation of job change and non-employment: Longitudinal data from the Whitehall II study. British Medical Journal, 1264–1269. | No quantitative analysis of both job insecurity AND unemployment towards the same health outcome |
|  | Gagin R and Shinan-Altman S (2012) Is Work Beneficial to Good Health?. Social Work in Health Care, 296–311. | No health outcome |
|  | Glozier N, Tofler GH, Colquhoun DM et al. (2013) Psychosocial risk factors for coronary heart disease: A consensus statement from the national heart foundation of Australia. Journal of Australia, 1–6. | No quantitative analysis of both job insecurity AND unemployment towards the same health outcome |
|  | Govender I (2009) Report on the 29th ICOH, International Congress on Occupational Health held in Cape Town from 22 to 27 March 2009. South African Family Practice. Retrieved from http://ovidsp.ovid.com/ovidweb.cgi?T=JS&CSC=Y&NEWS=N&PAGE=fulltext&D=emed9&AN=2009488172 | No quantitative analysis of both job insecurity AND unemployment towards the same health outcome |
|  | Hallsten L, Grossi G, Westerlund H (1999) Unemployment, labour market policy and health in Sweden during years of crisis in the 1990’s. International Archives of Occupational and Environmental Health. Retrieved from http://ovidsp.ovid.com/ovidweb.cgi?T=JS&CSC=Y&NEWS=N&PAGE=fulltext&D=emed4&AN=1999095109 | No quantitative analysis of both job insecurity AND unemployment towards the same health outcome |
|  | Ironson G (1992) Work, job stress, and health. Zedeck, Sheldon [Ed], 33–69. | No quantitative analysis of both job insecurity AND unemployment towards the same health outcome |
|  | Iversen L, Sabroe S, Damsgaard MT (1989) Hospital admissions before and after shipyard closure. British Medical Journal, 1073–1076. | No quantitative analysis of both job insecurity AND unemployment towards the same health outcome |
|  | Joelson L and Wahlquist L (1987) The psychological meaning of job insecurity and job loss: Results of a longitudinal study. Social Science & Medicine, 25(2), 179–182. | No quantitative analysis of both job insecurity AND unemployment towards the same health outcome |
|  | Jung Y, Oh J, Huh S et al. (2013) The Effects of Employment Conditions on Smoking Status and Smoking Intensity: The Analysis of Korean Labor & Income Panel 8th-10th Wave. PLoS ONE. | No health outcome |
|  | Krahn H, Lowe GS, Tanner J (1985) The social-psychological impact of unemployment in Edmonton. Journal of Public Health, 88–92. | No quantitative analysis of both job insecurity AND unemployment towards the same health outcome |
|  | Kupers T (1996) Men at work and out of work. Psychiatric Annals, 26(1), 29–32. | No quantitative analysis of both job insecurity AND unemployment towards the same health outcome |
|  | Kyriopoulos J and Tsiantou V (2010) The financial crisis and its impact on health and medical care. Archives of Hellenic Medicine, 834–840. | No quantitative analysis of both job insecurity AND unemployment towards the same health outcome |
|  | Levi L et al. (1984) The psychological, social, and biochemical impacts of unemployment in Sweden: Description of a research project. Journal of Mental Health, 18–34. | No quantitative analysis of both job insecurity AND unemployment towards the same health outcome |
|  | Lewis G and Sloggett A (1998) Suicide, deprivation, and unemployment: Record linkage study. British Medical Journal, 1283–1286. | No quantitative analysis of both job insecurity AND unemployment towards the same health outcome |
|  | Marchand A. et al. (2012) Psychological distress in Canada: The role of employment and reasons of non-employment. Int J Soc Psychiatry 58(6): 596–604. | Unemployment defined as reference category |
|  | Marmot MG (1999) Job insecurity in a broader social and health context. WHO regional publications, 1–9. | No quantitative analysis of both job insecurity AND unemployment towards the same health outcome |
|  | Marmot MG and & Bell R (2009) How will the financial crisis affect health? BMJ, 858–859. | No quantitative analysis of both job insecurity AND unemployment towards the same health outcome |
|  | Moulin JJ, Labbe E, Sass C et al. (2009) Job insecurity, unemployment and health: Results from the Health examination centers of the French General Health Insurance. Revue d’Epidemiologie et de Sante Publique, 141–149. | Job insecurity measured as part-time/full-time and permanent/non-permanent employment |
|  | Moynihan R (2012) Job insecurity contributes to poor health. BMJ (Clinical ed.). | No quantitative analysis of both job insecurity AND unemployment towards the same health outcome |
|  | Nakao M (2010) Work-related stress and psychosomatic medicine. BioPsychoSocial Medicine. | No quantitative analysis of both job insecurity AND unemployment towards the same health outcome |
|  | Noonan AE (2005) “At this point now”: older workers’ reflections on their current employment experiences. International journal of aging & human development, 61(3), 211–241. | No quantitative analysis of both job insecurity AND unemployment towards the same health outcome |
|  | Oliffe JL and Han CSE (2013) Beyond Workers’ Compensation: Men’s Mental Health In and Out of Work. American journal of men’s health. | No quantitative analysis of both job insecurity AND unemployment towards the same health outcome |
|  | Ostry AS, Hershler R, Kelly S et al. (2001) Effects of de-industrialization on unemployment, re-employment, and work conditions in a manufacturing workforce. BMC Public Health, 1–11. | No quantitative analysis of both job insecurity AND unemployment towards the same health outcome |
|  | Platt S (1984) Unemployment and suicidal behaviour: a review of the literature. Social science & medicine (1982), 19(2), 93–115. | No quantitative analysis of both job insecurity AND unemployment towards the same health outcome |
|  | Pockrandt C, Coder B, Lau K et al. (2007) Health behaviour and health-risk behaviour among job-seekers: A screening at an employment agency. Gesundheitswesen, 628–634. | No quantitative analysis of both job insecurity AND unemployment towards the same health outcome |
|  | Richardson L, Sherman SG, Kerr T (2012) Employment amongst people who use drugs: A new arena for research and intervention? Journal of Drug Policy, 3–5. | No quantitative analysis of both job insecurity AND unemployment towards the same health outcome |
|  | Rueda S, Raboud J, Rourke SB et al. (2012) Influence of employment and job security on physical and mental health in adults living with HIV: cross-sectional analyses. OM 6 (4). | Unemployment measured as non-employment |
|  | Rugulies R, Thielen K, Nygaard, E et al. (2010) Job insecurity and the use of antidepressant medication among Danish employees with and without a history of prolonged unemployment: a 3.5-year follow-up study. Journal of epidemiology and community health, 64(1), 75–81. | No health outcome |
|  | Schwarz P (2012) Neighborhood effects of high unemployment rates: Welfare implications among different social groups. Journal of Socio-Economics, 41(2), 180–188. | No quantitative analysis of both job insecurity AND unemployment towards the same health outcome |
|  | Schwefel D (1986) Unemployment, health and health services in German-speaking countries. Social Science and Medicine, 409–430. | No quantitative analysis of both job insecurity AND unemployment towards the same health outcome |
|  | Scuccimarra D and Speece D (1990) Employment outcomes and social integration of students with mild handicaps: The quality of life two years after high school. Journal of Learning Disabilities, 23(4), 213–219. | No quantitative analysis of both job insecurity AND unemployment towards the same health outcome |
|  | Stavrianakos K, Pachi A, Paplos K et al. (2013) Suicide attempts before and during the financial crisis in Greece. European Psychiatry. Retrieved from http://ovidsp.ovid.com/ovidweb.cgi?T=JS&CSC=Y&NEWS=N&PAGE=fulltext&D=emed11&AN=71172594 | No health outcome |
|  | Valty J, Frèal S, Nguyen KC et al. (1999) A multifactorial health risk questionnaire. International archives of occupational and environmental health, 72 Suppl, S6–7. | Unemployment measured as non-unemployment |
|  | Virtanen M, Kivimaki M, Ferrie JE, et al. (2008) Temporary employment and antidepressant medication: A register linkage study. Journal of Psychiatric Research, 221–229. | No quantitative analysis of both job insecurity AND unemployment towards the same health outcome |
|  | Virtanen P, Janlert U, Hammarstrom A (2011) Exposure to temporary employment and job insecurity: A longitudinal study of the health effects. Occupational and Environmental Medicine, 570–574. | No quantitative analysis of both job insecurity AND unemployment towards the same health outcome |
|  | Wahrendorf M, Blane D, Bartley M et al. (2013) Working conditions and mental health in older ages. Advances in Life Course Research 18(1):16-25. | Job insecurity measured as former unemployment experiences and fragmented working careers |
